# Evaluation of High-Performance Polyether Ether Ketone Polymer Treated with Piranha Solution and Epigallocatechin-3-Gallate Coating

**DOI:** 10.1155/2024/1741539

**Published:** 2024-04-08

**Authors:** Mohammed A. Alsmael, Aseel Mohammed Al-Khafaji

**Affiliations:** Department of Prosthodontics, College of Dentistry, University of Baghdad, Baghdad, Iraq

## Abstract

**Background:**

Dental implantation has become a standard procedure with high success rates, relying on achieving osseointegration between the implant surface and surrounding bone tissue. Polyether ether ketone (PEEK) is a promising alternative to traditional dental implant materials like titanium, but its osseointegration capabilities are limited due to its hydrophobic nature and reduced surface roughness.

**Objective:**

The aim of the study is to increase the surface roughness and hydrophilicity of PEEK by treating the surface with piranha solution and then coating the surface with epigallocatechin-3-gallate (EGCG) by electrospraying technique.

**Materials and Methods:**

The study includes four groups intended to investigate the effect of piranha treatment and EGCG coating: a control group of PEEK discs with no treatment (C), PEEK samples treated with piranha solution (P), a group of PEEK samples coated with EGCG (E), and a group of PEEK samples treated with piranha solution and coated with EGCG (PE). Surface roughness, wettability, and microhardness were assessed through statistical analysis.

**Results:**

Piranha treatment increased surface roughness, while EGCG coating moderated it, resulting in an intermediate roughness in the PE group. EGCG significantly improved wettability, as indicated by the reduced contact angle. Microhardness increased by about 20% in EGCG-coated groups compared to noncoated groups. Statistical analysis confirmed significant differences between groups in all tests.

**Conclusion:**

This study demonstrates the potential of EGCG coating to enhance the surface properties of PEEK as dental implants. The combined piranha and EGCG modification approach shows promise for improved osseointegration, although further vivo research is necessary. Surface modification techniques hold the key to optimizing biomaterial performance, bridging the gap between laboratory findings and clinical implementation in dental implantology.

## 1. Introduction

Dental implantation has become a routine procedure for replacing missing teeth and has become increasingly popular due to its high success rate and predictable outcomes. The success of dental implants relies on the establishment of a direct and stable interface between the implant surface and the surrounding bone tissue, known as osseointegration [1]. The osseointegration process depends on a number of factors, including the material and surface properties of the implant, as well as the biological response of the host tissue [2, 3].

Polyether ether ketone (PEEK) is a high-performance thermoplastic polymer that has attracted attention as a promising alternative to traditional dental implant materials such as titanium and its alloys [4, 5]. PEEK possesses excellent mechanical and biocompatible properties, including high strength, low density, and good chemical resistance [6]. However, PEEK has some limits in its osseointegration capabilities and its long-term clinical success as a dental implant material [7]. Several studies have reported that the limits in osseointegration of PEEK are related to its hydrophobic nature and low surface energy, which limit the adhesion and proliferation of bone cells on the implant surface [8]. To address this limitation, various surface modification techniques have been developed to improve the surface properties of PEEK and enhance its osseointegration ability. Among these techniques are surface modification techniques such as laser treatment and acid conditioning [9], one of the most popular used acid treatments is piranha solution and it has been widely used on PEEK for multiple purposes starting from increasing wettability of the surface and reaching to increase the bonding ability of different coating materials [10]. In a previous article, we have investigated the effect of using piranha treatment alone on some properties of PEEK [11]. On the other hand, the coating of PEEK with bioactive molecules has emerged as a promising approach for enhancing its osseointegration ability [12]. The use of bioactive coatings can modify the surface chemistry of PEEK to promote the adhesion, proliferation, and differentiation of bone cells, as well as regulate the inflammatory response at the implant-tissue interface [13].

Epigallocatechin-3-gallate (EGCG) is a major polyphenol found in green tea that has been shown to possess a wide range of biological activities, including antioxidant, anti-inflammatory, and antimicrobial properties [12]. In addition, EGCG has been reported to promote bone formation and inhibit osteoclast activity, making it a promising candidate for improving the osseointegration of dental implants [14]. Several studies have investigated the effects of EGCG coating on dental and biomedical materials like titanium or other types of alloys and even some polymers [15–18]. However, to the best of our knowledge, no studies have investigated the effects of pure EGCG coating on the properties of PEEK.

In this study, the effects of piranha surface treatment and EGCG coating on the surface properties of PEEK were investigated in vitro. It was hypothesized that the EGCG coating combined with piranha surface treatment will enhance the hydrophilicity and surface energy of PEEK. This improvement may contribute to developing PEEK dental implants with improved bone formation around them. Our findings may provide a new approach for developing PEEK-based dental implants with improved clinical outcomes.

## 2. Materials and Methods

### 2.1. Sample Preparation

**Figure 1:**
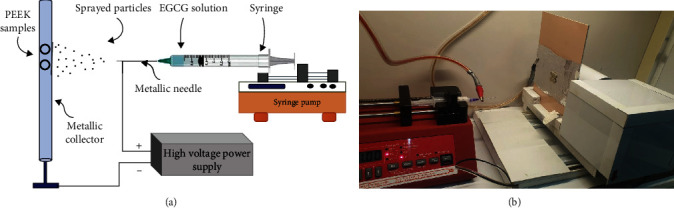
(a) Illustration of the electrospraying system and the arrangement of PEEK samples and EGCG solution in the system. (b) Actual picture of the electrospraying system with PEEK samples on the metallic collector and EGCG solution in the syringe with needle attached to the clip connected to the positive charge of a high power supply.

**Figure 2:**
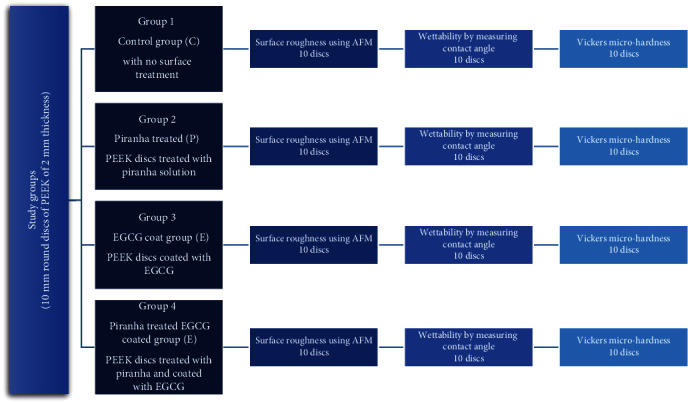
Flowchart showing the study design and different study groups.

10 mm round discs of PEEK with a thickness of 2 mm were used [19]. The PEEK samples were obtained from a commercial supplier (Energetic Industry Co., China) and were verified to be of consistent quality and dimensions. Smoothening of the surface was done using silicon carbide paper starting from 500 up to 2400 grit; in order to have a uniform smooth surface, this process was done using a rotating polishing machine at a speed of 250 rounds per minute (rpm), for two minutes of each step. Ultrasonic cleaner was used to clean the discs after the polishing process, first ethanol then by distilled water for 15 minutes and 10 minutes, respectively. In the last step, polished discs were left to dry at room temperature for 15 minutes. Four groups each containing 10 discs of PEEK were prepared for each test of the tests conducted, as follows:

- Group 1: control group (C). This group served as the baseline and did not undergo any treatment
- Group 2: piranha solution treatment group (P). PEEK samples in the group were treated with piranha solution, which was prepared by mixing sulfuric acid (98% concentration, Beckson Co., USA) and hydrogen peroxide (30% concentration, Emsure Co., Germany) in a specific ratio of 3 : 1 (*v*/*v*) to achieve the desired concentration. The PEEK samples were immersed in a glass beaker filled with freshly prepared piranha solution for 1 minute [10, 20–22]. After removal of the samples from the glass container, they were cleaned ultrasonically in distilled water and in isopropyl alcohol each for 10 minutes and then they were left to dry at room temperature for 15 minutes [23]
- Group 3: EGCG coating group (E). PEEK samples were coated with epigallocatechin-3-gallate (EGCG) by using electrospraying. EGCG powder (purity >98%, Hangzhou Hyper Chemicals Limited, China) was dissolved in deionized water to prepare a 2 wt% EGCG solution. The PEEK samples were then coated with the EGCG using a custom-made electrospray system with a spray voltage of 20 kV and a flow rate of 0.2 mL/h. PEEK samples appear in Figure 1 in the electrospraying system attached to the metal collector while the solved EGCG mixture is in the syringe with its nozzle attached clip which is connected to the positive charge of a high power supply. This design was made according to multiple previous papers and articles [24–27]
- Group 4: piranha solution treatment and EGCG coating group (PE). PEEK samples in this group underwent both piranha solution treatment and EGCG coating. First, the PEEK samples were treated with piranha solution, which was prepared by mixing sulfuric acid (98% concentration, Beckson Co., USA) and hydrogen peroxide (30% concentration, Emsure Co., Germany) in a specific ratio of 3 : 1 (*v*/*v*) to achieve the desired concentration. The PEEK samples were immersed in the piranha solution for 1 minute; after removal of the samples from the glass container, they were cleaned ultrasonically in distilled water and in isopropyl alcohol each for 10 minutes and then they were left to dry at room temperature for 15 minutes similar to treatment described in Group 2. Then, the samples were coated with EGCG solution using the same electrospray system and parameters as described in Group 3. The study design is also shown as a flowchart in Figure 2

### 2.2. Characterization of Samples

#### 2.2.1. Surface Roughness

The surface roughness of the PEEK samples was measured using an atomic force microscope (AFM, NaioAFM, Nanosurf Switzerland). The AFM was calibrated according to the manufacturer's instructions, and a 10 *μ*m × 10 *μ*m scan size was used for all samples. Roughness values (Ra) were calculated by the software of the AFM and reported in nanometers [28]. Surface roughness analysis by AFM was chosen in this study due to its precise imaging quality and great ability to analyse, revealing vital details about topography, which affects osseointegration, stability, and biocompatibility for improved biomedical outcomes [29, 30].

#### 2.2.2. Wettability

The wettability of the PEEK samples was evaluated by measuring the contact angle using a contact angle goniometer (Creating Nano Technologies Inc., Taiwan). A droplet of deionized water (10 *μ*m) was released from a syringe onto the horizontally positioned sample. The droplet was allowed to disperse on the sample for 30 seconds, and then an image was taken. The contact angles were measured using computer software, and each specimen was measured in four different areas, and the average of readings was reported for each specimen [19]. Wettability was chosen to be studied in this study since it gauges how well implants interact with fluids, influencing cell adhesion, protein adsorption, and overall biocompatibility, crucial for successful implant integration [31].

#### 2.2.3. Microhardness

The microhardness of the PEEK samples was evaluated using the Vickers hardness testing method. This involved utilizing a Vickers hardness tester (TMTeck Inc, China) to perform the measurements. The tester's indenter was pressed into the surface of the PEEK sample, at a constant load of 100 g and loading time of 15 seconds. An average of six readings were reported as the microhardness of the samples. The resulting impressions were then measured diagonally, and the hardness value was calculated by taking the average of the six readings. This value provides information about the material's resistance to indentation and deformation [32, 33]. Out of concern for potential surface hardness alterations due to treatment and coating, microhardness testing was selected to assess any changes occurring in the surface of the PEEK material.

### 2.3. Statistical Analysis

All experiments were performed in triplicate, and the results were expressed as mean ± standard deviation (SD). Statistical analysis was performed using analysis of variance (ANOVA) followed by post hoc Tukey's test for multiple comparisons. A *P* value less than 0.05 was considered statistically significant.

## 3. Results

### 3.1. Roughness

Assessment of normality assumption was carried out using the Shapiro-Wilk test. The resulting *P* values for the C, P, E, and PE groups are as shown in Table 1. Based on these values, there is insufficient evidence to reject the hypothesis of normality. Therefore, it can be inferred that the data is normally distributed across all groups.

**Figure 3:**
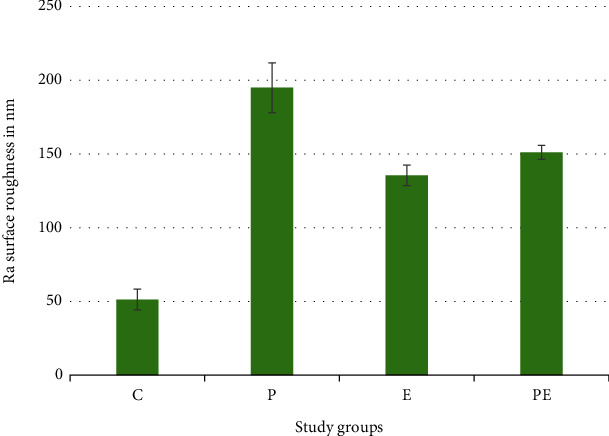
Bar chart showing average values and standard deviation of roughness values for control and experimental groups.

Surface roughness was evaluated by comparing the Ra values obtained from AFM tests conducted on various groups. The control group (C) exhibited the lowest average roughness at 51.37 nm. In contrast, the group treated with piranha without any coating (P) demonstrated the highest roughness value at 194.89 nm; for the other groups (E and PE), the roughness was intermediate between these two values. It can be seen in Figure 3 and Table 2.

Concerning the descriptive statistics of these findings, the one-way ANOVA's *F*-test indicates a remarkably significant variation in surface roughness (*P* value = 0.000) among the four categorized groups (C, P, E, and PE), as presented in Table 3.

The analysis using Tukey's multiple comparison test revealed that significant differences between groups C and P, C and E, C and PE, P and E, and P and PE were highly significant; conversely, the comparison between groups E and PE displayed a nonsignificant difference, as shown in Table 4.

### 3.2. Wettability

Assessment of normality assumption was also carried out for wettability using the Shapiro-Wilk test. The resulting *P* values are shown in Table 1. Based on these values, there is insufficient evidence to reject the hypothesis of normality. Therefore, it can be inferred that the data is normally distributed across all groups.

**Figure 4:**
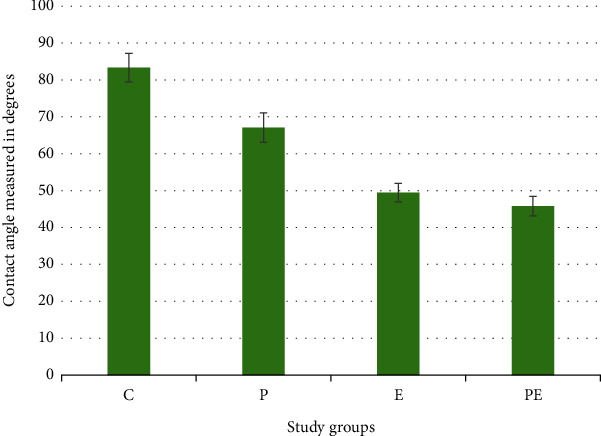
Bar chart showing average values and standard deviation of wettability by measuring contact angle values for control and experimental groups.

**Figure 5:**
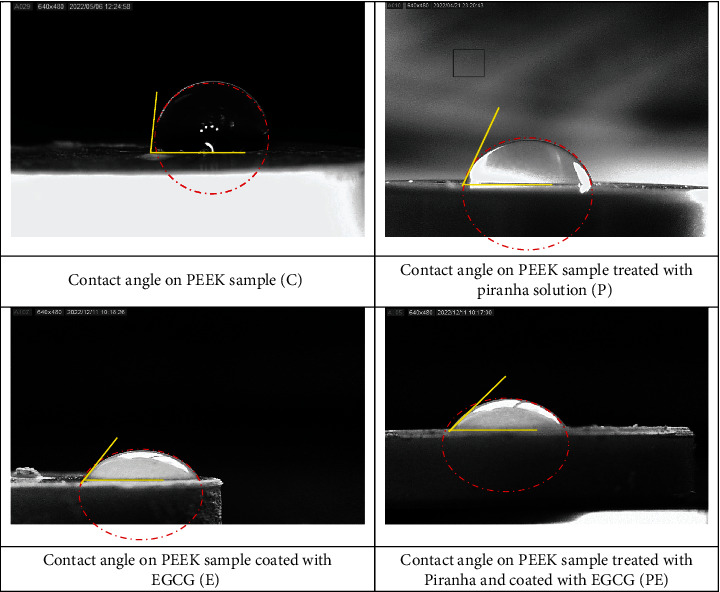
Wettability test by measuring the contact angle of different experimental groups.

The evaluation of sample wettability across diverse groups was carried out through contact angle measurement. The mean contact angle for the control group (C) registered the highest value at 83.34°, marking the most elevated angle among all groups. Conversely, the PE group exhibited the lowest value with an average contact angle of 45.80°. The remaining groups, P and E, demonstrated contact angles of 67.08° and 49.46°, respectively. These findings are presented in Figure 4 and Table 5. It is important to highlight that this approach is quantitative in nature, as a decrease in the contact angle signifies enhanced wettability, while an increase indicates the opposite.  Figure 5 shows a sample of each group. A decrease in contact angle is noticeable in the pictures, and it indicates an increase in wettability compared to the control group.

Concerning the descriptive statistics of these findings, the one-way ANOVA's *F*-test indicates a remarkably significant variation in surface roughness (*P* value = 0.000) among the four categorized groups (C, P, E, and PE), as presented in Table 6.

The utilization of Tukey's multiple comparison test revealed notable distinctions among the following group pairs: C and P, C and E, C and PE, P and E, and P and PE. These differences were highly significant. In contrast, the comparison between groups E and PE indicated an absence of significant disparity. These outcomes are presented in detail in Table 7.

### 3.3. Hardness Vickers

Assessment of normality assumption was carried out for the hardness test using the Shapiro-Wilk test. The resulting *P* values for the C, P, E, and PE groups are presented in Table 1. Based on these values, there is insufficient evidence to reject the hypothesis of normality. Therefore, it can be inferred that the data is normally distributed across all groups.

**Figure 6:**
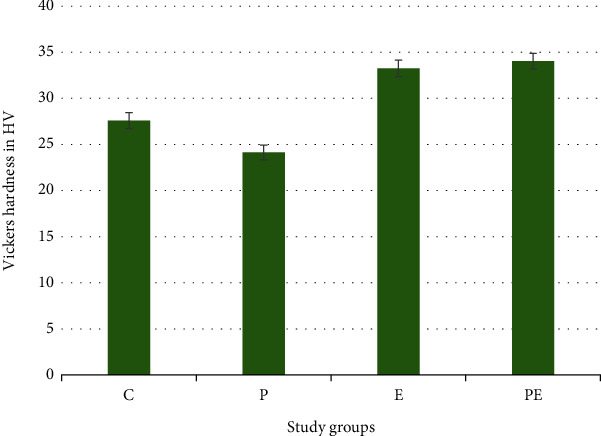
Bar chart showing average values and standard deviation of microhardness values for control and experimental groups.

The evaluation of hardness across different groups was done by using a Vickers hardness instrument with a diamond indenter. The mean for the control group (C) registered a value of 27.587 HV. The lowest value was recorded in group P that was without coating, and it was 24.134 HV. The remaining groups, E and PE, demonstrated hardness values of 33.242 HV and 34.031 HV, respectively. These findings are presented in Figure 6 and Table 8.

Regarding descriptive statistics of these findings, the one-way ANOVA's *F*-test indicates a remarkably significant variation in surface roughness (*P* value = 0.000) among the four categorized groups (C, P, E, and PE), as presented in Table 9.

Tukey's multiple comparison test revealed high distinctions among the following group pairs: C and P, C and E, C and PE, P and E, and P and PE. These differences were highly significant. In contrast, the comparison between groups E and PE indicated an absence of significant disparity. These outcomes are presented in detail in Table 10.

## 4. Discussion

Polyether ether ketone (PEEK), a high-performance thermoplastic polymer, has emerged as a promising candidate for dental implants due to its exceptional mechanical properties and biocompatibility [34, 35]. However, its hydrophobic nature and low surface energy have presented challenges in achieving optimal osseointegration [36]. These limitations were the focal point of this study, which aimed to investigate the effects of piranha surface treatment and epigallocatechin-3-gallate (EGCG) coating on the surface properties of PEEK dental material.

The findings of this study corroborate the growing body of evidence suggesting that surface modification techniques hold the key to unlocking the full potential of biomaterials [37, 38]. The initial step in understanding the impact of EGCG coating on PEEK surfaces was the evaluation of surface roughness, a crucial factor in cell adhesion and proliferation. The increase in roughness observed after piranha treatment aligns with previous studies that have highlighted the etching effect of this solution [10, 39]. Interestingly, after applying the EGCG coating, the surface roughness reached an intermediate value between the control group and the piranha-treated group. This dual modification approach offers a unique opportunity to create a topography that could potentially encourage cell attachment while also promoting a more secure anchorage of the implant within the surrounding bone tissue.

The improvement in wettability is another essential outcome of this study. The wetting behavior of a material has a profound impact on cell responses and protein adsorption [20]. EGCG, known for its hydrophilic nature, significantly reduced the contact angle values on the coated samples [40]. The reduction in contact angle suggests that the EGCG-coated surfaces are more prone to wetting, which has been linked to enhanced cell attachment and better biological responses [41]. The implications of this finding are significant, as improved wettability may foster a favorable microenvironment at the implant-tissue interface, thereby accelerating the osseointegration process.

The microhardness measurements yielded interesting results. While the EGCG coating appeared to enhance the hardness of the PEEK surface, the extent of this improvement was less pronounced compared to the changes observed in roughness and wettability. The underlying mechanism behind this phenomenon needs further investigation. It is possible that the deposition of EGCG particles on the surface might have contributed to reinforcing the material, leading to the observed changes in hardness, and multiple other studies showed an increase in microhardness after coating with different materials [42, 43]. Another study also found an increased microhardness of dental cements after EGCG coating [44]. However, the details of this interaction necessitate deeper exploration.

The success of implantation goes beyond surface properties; it extends into the biological responses and long-term clinical outcomes. EGCG, the star of this study, has attracted attention for its diverse biological activities, ranging from antioxidant and anti-inflammatory properties to bone-stimulating effects [45, 46]. The promotion of bone formation and inhibition of osteoclast activity make EGCG a potential game-changer in the field of dental implants. While previous research has explored the effects of EGCG on various implant materials, including titanium implants [15] and bone graft materials [47], the novelty of this study lies in its focus on PEEK—a material with unique challenges and opportunities and also on the novel coating technique for EGCG.

While the in vitro results provide compelling evidence for the potential of EGCG-coated PEEK implants, the dynamic and multifaceted environment within the human body poses unique challenges. In vivo studies are imperative to assess the biological responses to EGCG-coated PEEK implants within the internal systems of living organisms. Long-term stability, biomechanical interactions, and immunological responses should all be evaluated to measure the full spectrum of effects that this modification may exert.

Furthermore, the practicality and feasibility of the proposed EGCG-coating technique should be evaluated. The electrospray method employed in this study offers precision and control over the coating process [48, 49], and it is the first time to be used with EGCG in any substrate. Additionally, the potential release of EGCG over time and its impact on the surrounding tissues should be explored as this technique is traditionally used as a drug delivery method which may increase the benefits of using EGCG on the surrounding bone [50, 51].

The study presented here contributes valuable insights into the realm of dental implant materials. The surface modification approach, especially the combination of piranha treatment and EGCG coating, holds great promise in overcoming the limitations of PEEK as a dental implant material. The enhanced roughness, improved wettability, and potential strengthening observed through microhardness measurements collectively suggest that EGCG-coated PEEK implants could offer an exciting avenue for achieving improved osseointegration and long-term success. The synthesis of this particular combination involving the application of pure epigallocatechin gallate (EGCG) onto polyether ether ketone (PEEK) substrate represents a novel achievement not documented previously in scientific literature, to the best of our knowledge. However, the path from these laboratory findings to clinical implementation is complex and requires meticulous investigation, especially regarding the biocompatibility and any effects on surrounding vital structures. This study underscores the importance of interdisciplinary collaborations and translational research efforts to bridge the gap between the bench and the bedside, ultimately improving the quality of life for patients seeking dental implant solutions.

## 5. Conclusion

This study investigated the potential enhancement of surface properties of polyether ether ketone (PEEK) after treating the surface with piranha solution and coating with epigallocatechin gallate (EGCG). The findings underscore the significance of surface modification techniques in improving biomaterial performance. The dual modification approach involving piranha treatment and EGCG coating led to increased roughness, improved wettability, and potential strengthening. These modifications offer promising horizons for fostering enhanced osseointegration. However, clinical translation demands serious in vivo investigations to validate the observed effects within the complex biological environment.

## Figures and Tables

**Table 1:** Shapiro-Wilk test for all groups in AFM, wettability, and roughness.

Tests	Group C		Group P		Group E		Group PE	
	*P* value	Passed	*P* value	Passed	*P* value	Passed	*P* value	Passed
AFM	0.167 NS	Yes	0.541 NS	Yes	0.345 NS	Yes	0.845 NS	Yes

Wettability contact angle test	0.555 NS	Yes	0.432 NS	Yes	0.342 NS	Yes	0.598 NS	Yes

Hardness (Vickers)	0.678 NS	Yes	0.338 NS	Yes	0.753 NS	Yes	0.134 NS	Yes

**Table 2:** Data describing roughness (AFM) results.

	Mean (nm)	Standard deviation	Minimum (nm)	Median (nm)	Maximum (nm)
C	51.37	14.0871	32.77	47.13	69.827
P	194.89	33.6454	148.17	198.42	241.56
E	135.45	13.8431	108.7	139.3	154.1
PE	151.02	9.4608	138.2	149.7	170.2

**Table 3:** One-way ANOVA test of roughness by AFM.

Test	Within groups			Between groups			*F*	Sig.
	Sum of squares	Df	Mean square	Sum of squares	Df	Mean square		
Roughness	14504.41	36	402.9002	108244.1	3	36081.35	89.554	0.000 (HS)

**Table 4:** Tukey's test of multiple comparisons for different groups of roughness test results.

Tukey's HSD test					
Group pairs	Difference	Standard error	*Q* score	*P* value	Sig.
C vs. P	143.5209	6.347442	22.61082	< 0.00001	S
C vs. E	84.0769	6.347442	13.24579	< 0.00001	S
C vs. PE	99.6469	6.347442	15.69875	< 0.00001	S
P vs. E	59.444	6.347442	9.365032	< 0.00001	S
P vs. PE	43.874	6.347442	6.912076	0.00012	S
E vs. PE	15.57	6.347442	2.452957	0.32128	NS

**Table 5:** Data describing wettability by measuring contact angle results.

	Mean	Standard deviation	Minimum	Median	Maximum
C	83.342°	3.8684	78.34°	83.65°	89.69°
P	67.082°	3.9975	62.08°	66.1°	74.53°
E	49.458°	2.519	45.6°	49.18°	55.13°
PE	45.804°	2.623	41.18°	46.515°	49.4°

**Table 6:** One-way ANOVA test of wettability by measuring the contact angle.

Test	Within groups			Between groups			*F*	Sig.
	Sum of squares	Df	Mean square	Sum of squares	Df	Mean square		
Wettability	397.5291	36	11.04248	8995.812	3	2998.604	271.5518	0.000 (HS)

**Table 7:** Tukey's test of multiple comparisons for different groups of wettability results.

Tukey's HSD test					
Group pairs	Difference	Standard error	*Q* score	*P* value	Sig.
C vs. P	16.26	1.050832	15.47345342	< 0.00001	S
C vs. E	33.884	1.050832	32.24492593	< 0.00001	S
C vs. PE	37.538	1.050832	35.72217062	< 0.00001	S
P vs. E	17.624	1.050832	16.77147251	< 0.00001	S
P vs. PE	21.278	1.050832	20.24871721	< 0.00001	S
E vs. PE	3.654	1.050832	3.477244698	0.08406	NS

**Table 8:** Data describing wettability by measuring contact angle results.

	Mean in (HV)	Standard deviation	Minimum (HV)	Median (HV)	Maximum (HV)
C	27.587	0.9038	25.92	27.88	29.04
P	24.134	0.8429	22.77	23.84	25.83
E	33.242	0.8815	32.00	33.135	34.56
PE	34.031	0.945	32.12	34.375	35.03

**Table 9:** One-way ANOVA test of Vickers hardness test results.

Test	Within groups			Between groups			*F*	Sig.
	Sum of squares	Df	Mean square	Sum of squares	Df	Mean square		
Hardness (Vickers)	28.7781	36	0.799392	667.3904	3	222.4635	278.291	0.000 (HS)

**Table 10:** Tukey's test of multiple comparisons for different groups of Vicker's hardness test results.

Tukey's HSD test					
Group pairs	Difference	Standard error	*Q* score	*P* value	Sig.
C vs. P	3.453	0.282735	12.21285	< 0.00001	S
C vs. E	5.655	0.282735	20.00106	< 0.00001	S
C vs. PE	6.444	0.282735	22.79166	< 0.00001	S
P vs. E	9.108	0.282735	32.21391	< 0.00001	S
P vs. PE	9.897	0.282735	35.00451	< 0.00001	S
E vs. PE	0.789	0.282735	2.790599	0.21691	NS

## Data Availability

The data that support the findings of this study are available from the corresponding author upon reasonable request.
